# Excision of the Clavicle for Osteo-Sarcoma

**Published:** 1829-04-01

**Authors:** 


### XLIII.

### Exsection of the Clavicle for Osteo-sarcoma.*

Valentine Mott has added another laurel to the wreath which already encircles his brows, and equalled, if not crowned his former masterly and splendid operations, by excising the clavicle for osteo-sarcoma. This bold proceeding is highly deserving of being placed upon record in the journals devoted to medical and surgical science on both sides the Atlantic, and we gladly transfer the principal points of the case to our pages.

W. Yates, a plethoric young man, ætat. 19, discovered in the early part of February, 1828, a tumour about the size of a pigeon's egg, hard, and immoveable, in the left clavicle, unattended by pain or discolouration of the skin. His health was good, and no obvious or satisfactory cause could be given for its appearance. He consulted a physician, who thought it an encysted tumour, and applied warm salt water, blisters, poultices, a seton, and escharotics, without any good effect, and in May the patient sailed from Charleston, where he was, to New York, and consulted Professor Mott in the latter city. At this time the tumour was conical, four inches in diameter at its base, incompressibly hard, and firmly attached to the anterior portion of the clavicle. The apex of the tumour was covered with luxuriant fungous granulations, from which profuse bleedings at times took place. The patient being anxious for an operation on account of the rapid increase of the tumour, and willing to undergo the risks and uncertainty candidly pointed out as attending it, the 17th of June was chosen for its performance. The steps we find it impossible to describe in any but the talented Professor's own words.

"He was placed upon a table, with his shoulders a little elevated, inclining to the left side. Assisted by Dr. Barrow, Dr. Proudfoot, and Dr. A. E. Hosack, in the presence of Drs. Hull, Storer, Leveridge, Pratt, and a number of my pupils, the following operation was performed:—

"An incision was commenced over the articulation of the clavicle, with the sternum, and carried in a semicircular direction, as close to the fungus projections as the sound integuments would admit of, until it terminated on the top of the shoulder, near the junction of the clavicle, with the acromion process of the scapula. This incision exposed the fibres of the pectoralis major, which was divided as near the tumour as possible; in accomplishing this, as well as the first incision, arteries sprung in every direction, and required ligatures. A number of large branches of veins, under this muscle, emitted blood freely, and required to be tied.

"In conducting the incision through the pectoral muscle, towards the scapular extremity of the clavicle, care was taken to avoid the cephalic vein, as it passes between this and the deltoid mucle. A small portion of the latter muscle was detached from the clavicle, which readily allowed the vein to be drawn outward towards the shoulder.

"On attempting to pass the forefinger under the vein and deltoid to the lower edge of the clavicle, it was found impracticable, as the hard osseous part of the tumour extended beyond this point, and was completely in contact with the coracoid process of the scapula.

"Finding it impossible, from the size of the tumour and its proximity to the coracoid process, to get under the clavicle in this direction, an incision was made from the outer edge of the external jugular vein, over the tumour, to the top of the shoulder. After dividing the skin, platysma myoides, and a portion of the trapezius muscle, a sound part of the clavicle was laid bare at a point nearer the acromion than a line with the coracoid process; a steel director, very much curved, was now cautiously passed under the bone from above; which, from the firm bony state of the tumour at this part, had a considerable obliquity outwards. Great care was taken to keep the instrument in close contact with the under surface of the bone. The depth of the bone from the surface, rendered it somewhat difficult to accomplish this safely: an eyed-probe, similarly curved, conveyed along the groove of the director a chain saw, which, when moved a little, showed that nothing intervened between

* American Journal of the Medical Sciences, No. V.

it and the bone; the clavicle was then readily sawed through.

"The dissection was now continued along the under surface of the tumour, below the pectoralis major; here a number of very large arteries and veins required tying. The first rib being next exposed under the sternal extremity of the clavicle, the costo-clavicular or rhomboid ligament was divided, and the joint opened from the lower part. This gave considerable mobility to the diseased mass, and encouraged us to believe that its complete removal would be practicable.

"By means of a double hook and elevator, with the assistance of our strong and very broad spatulas, properly curved, we were enabled to elevate a little the sawed end of the clavicle. After loosening the parts about it, by keeping close to the tumour, we wished to discover the subclavius muscle, as it is inserted in the bone about this situation; but it could not be seen, as it was incorporated with the diseased mass. Had this muscle been found, the separation of the tumour would have been much less difficult and tedious, as, by keeping above it, the subclavian vein is of course protected. The origin of this muscle, from the cartilage of the first rib, was seen and divided, but it was almost immediately obliterated in the tumour.

"Continuing the removal of the tumour at the upper and outer part, the omo-hyoideus was found lying under it, which we exposed from where it passes under the mastoid muscle, to near its origin from the superior costa of the scapula. In separating the tumour from the cellular and fatty structure, between the omo-hyoid muscle and the subclavian vessels, a number of large arteries were divided, which bled freely, and particularly a large branch from the inferior thyroidal.

"The anterior part of the upper incision was now made from the sternal end of the clavicle, and carried over the tumour, until it met the other at the external jugular vein. After cutting through the platysma myoides, this vein was carefully separated from the surrounding parts, and two fine ligatures passed beneath it, and tied a short distance from each other; the vein was then cut between the ligatures.

"The clavicular part of the sterno-cleido-mastoideus was next divided, about three inches above the clavicle in the direction of this incision. The deep-seated fascia of the neck being now exposed, the mastoid muscle, and the diseased mass, were very cautiously separated from it, until the anterior scalenus was exposed.

"The subclavian vein, from the edge of the scalenus anticus to the coracoid process, was so firmly adherent to the tumour, as to lead me at one moment to believe that the coats of the vein were so intimately involved in the diseased structure, as to render the complete removal of the morbid part utterly impracticable. By the most cautious proceeding, however, alternately with the handle and blade of the knife, we finally succeeded in detaching the tumour, without the least injury to the vein. This part of the operation was attended with peculiar danger and difficulty. At every cut either an artery or vein would spring, and deluge the parts until secured by ligatures. Besides several large veins, the external jugular was so situated in the midst of the bony mass, as to require two more ligatures in this place, near to the subclavian, and it was again divided in the interspace. Near the sternal end of the clavicle, a large artery and vein required tying; they were considered as branches of the inferior thyroidal artery and vein.

"From having cut through the clavicular portion of the mastoideus muscle, obliquely upwards and outwards a little above the tumour, we were enabled, by turning this down, and keeping close to the fascia profunda, to detach the tumour from over the situation of the thoracic duct and junction of the internal jugular and left subclavian, without the least injury to these important parts.

"To reach the lower part of the tumour as it extended upon the thorax, it was necessary to separate the pectoralis major in a line with the fourth rib, and to make a transverse incision two inches in length through the integuments and muscles at about its centre. The incision upon the neck extended from the sterno-clavicular junction in a semicircular direction, to within an inch of the thyroid cartilage and base of the lower jaw, and two inches from the lobe of the ear, and terminated near the junction of the clavicle and scapula.

"The fungous and bleeding character of the apex of the tumour implied that it was freely supplied with vessels. The discharge of blood was so free at every step of the operation, that about forty ligatures were applied. It was estimated that the patient lost from sixteen to twenty ounces of blood.

"All the parts now presenting a healthy appearance, the ligatures were cut close to the knots, and the cavity of the wound filled with lint. Long strips of adhesive plaster were applied to prevent the edges of this extensive wound from further retracting; a light compress, a single-headed roller loosely applied around the chest and shoulders, completed the dressing.

"He was placed in bed upon his back, inclining a little to the right side, with the head considerably elevated, whilst the left shoulder and arm were supported by a pillow."

The particulars of the after-treatment, as well as the diurnal details, present nothing worthy of notice; suffice it to say, that no symptoms of an untoward description occurred, and the patient, in the middle of August, was able to leave the city of New York on an excursion to the springs at Saratoga. In September he returned, enjoying better health than he had ever done before.

"The tumour is about the size of a man's double fists, or of a circumference just to allow me to grasp it with my fingers fully extended. It consists of a bony cup, incompressibly hard at all parts, except superiorly and inferiorly to a small extent. From an opening of an elliptical shape at the upper part, protruded a bleeding fungus of the size and shape of half a hen's egg. At the under surface, as it lay upon the great subclavian vessels, the bony character is less manifest; the structure about the centre particularly appearing to be cartilaginous or semi-osseous. This bony enlargement occupies the clavicle from the sternal articulation to within half an inch perhaps of the acromial extremity. From the motion which can be given to each end of the clavicle, the natural structure of the bone seems to be entirely destroyed.

"The operation far surpassed in tediousness, difficulty, and danger, any thing which I have ever witnessed or performed. It is impossible for any description which we are capable of giving, to convey an accurate idea of its formidable nature. The attachment of the morbid mass to the important structures of the neck and shoulder of *the left side* and to so great an extent, is sufficient to indicate its magnitude and difficulty.

"The extensive nature of this operation, led us to take the precaution of securing the external jugular with a double ligature and dividing it between them. Though in operating upon the neck we have several times cut these veins without any unpleasant consequences, we however think we have witnessed almost fatal effects from the division of a large vein, and the admission of air into the circulation.

"The case of Baron Dupuytren's, in which a young woman suddenly died under an operation, from the division of a large vein in the neck, whilst he was engaged in removing the tumour, contributed with my own experience, to make me take the precaution of previously tying the vien in this operation.

"In an attempt which I made to remove the parotid gland in an enlarged and scirrhous state, the facial vein, where it passes over the base of the lower jaw, was opened in dissecting the integuments from the tumour, in the early stage of the operation, before a single artery was tied. At the instant this vessel was opened, the attention of all present was arrested by the gurgling noise of air passing into some small opening. The breathing of the patient immediately became difficult and laborious, the heart beat violently and irregularly, his features were distorted, and convulsions of the whole body, soon followed to so great an extent as to make it impossible to keep him on the table. He lay upon the floor in this condition for near half an hour, as all supposed in *articulo mortis*. As the convulsions gradually left him, his mouth was permanently distorted, and complete hemiplegia was found to have ensued. An hour and more elapsed before he could articulate, and it was nearly a whole day before he recovered the use of his arm and leg. From a belief that these effects arose from the admission of air into the blood-vessels, which was not doubted by any person present, I instantly called to mind a set of experiments, which I made some twenty years since upon dogs, by blowing air into the circulation, by inserting a blow-pipe into a large superficial vein upon the thigh, and was forcibly struck with the similarity of result.

"To the extraordinary composure of mind which our patient manifested, is to be attributed in a great measure his undisturbed and speedy recovery."

"No difficulty attended keeping his shoulder in a proper position by the use of the common apparatus for fractured clavicle. With this he walked about without any inconvenience, after four weeks elapsed, and two months from the time of the operation, he was able to discontinue the sling, and by means of an apparatus contrived by Mr. James Kent, a most ingenious and inventive artist, to supply the want of clavicle, he was so fitted as to have his shoulder in its proper position, at the same time that the full motion of his arm was preserved."

The case altogether does no discredit to American surgery, high as it at present most deservedly stands.

